# Rabies in Sheep, with Inoculation Experiments on Rabbits

**Published:** 1897-05

**Authors:** A. W. Clement, William Royal Stores

**Affiliations:** State Veterinarian, Maryland; Bacteriologist to the Health Department and Lecturer on Bacteriology in the Baltimore Medical College


### THE JOURNAL
OF
### COMPARATIVE MEDICINE AND VETERINARY ARCHIVES.

VOL. XVIII. *MAY*, 1897. No. 5.

### RABIES IN SHEEP, WITH INOCULATION EXPERIMENTS ON RABBITS.

BY A. W. CLEMENT, V.S.,
STATE VETERINARIAN, MARYLAND,

AND

WILLIAM ROYAL STOKES, M.D.,
BACTERIOLOGIST TO THE HEALTH DEPARTMENT AND LECTURER ON BACTERIOLOGY IN THE BALTIMORE MEDICAL COLLEGE.

IN the month of July, 1896, a gentleman from Mullikens, Prince George County, reported "a strange disease" among his cattle and sheep, from which several had died and of which others were ill. I visited the farm the day after receiving notice of the outbreak, and heard from the owner the history of the outbreak and his observation of the symptoms.

He had twenty young cattle running in a field with abundance of shade, water, and grass, and in fine order. Two steers died first, within a day or two of each other, after a short illness. Ten days later a third steer was attacked similarly and died, and a month after the first steer was taken sick a fourth one died. When I arrived I found the fifth steer dead, but none of the remaining number appeared sick.

The symptoms as described by the owner were as follows: "At first they began to lose flesh rapidly. A few days afterward they isolated themselves and remained perfectly quiet. They would neither eat nor drink. After remaining in this condition two or three days they lost, to a certain extent, control of their hind legs and staggered as they walked. They then became very vicious and would attack any object, and bellowed constantly until they died."

The autopsy which I made upon the steer found dead showed no lesions whatever, though a thorough examination (excepting the brain and spinal cord) was made. I could make no diagnosis, and so informed the owner.

It was stated that a mad dog had passed through the neighborhood about Easter; but I, unfortunately, did not pay much attention to the statement, as it was some time before the hydrophobia excitement in Baltimore, and I had not seen a case of rabies since I was in Paris, ten years before. I did not believe that the dog had rabies.

About January 1st the same gentleman wrote me that he had lost several sheep on the same pasture where the cattle fed. Under date of January 4th he wrote that eleven had died, and he had discovered another lamb affected that afternoon. He described the symptoms as follows: "The face swells on both sides just below the nose, and one of the animals looked first to one side and then the other, as if in pain. They do not appear to suffer much, as a rule, and some of them eat a little. They all have the same vicious propensities from the commencement of the attack, which increase as the disease develops. As a general thing, they live about four days." As to the cause, the gentleman cited the observation of a neighbor, who said that he saw a dog chasing his own sheep about ten days before; since then he had lost three. He further wrote: "A dog has not been in this flock since last Easter, when three were killed, and some of the lambs which have since been affected were not born then."

In answer to this letter I asked the owner to ship me by express, well boxed, a live sheep affected with the disease, whatever it might be, for the purpose of making a thorough clinical observation and also that the condition might be most favorable for autopsy, cultures, and inoculations. Accordingly, on the morning of January 6th there arrived in Baltimore by express a full-grown ewe. This animal was secured in a crate. When not disturbed at all it would lie or stand perfectly quiet, except occasionally, when it would butt its head against the slats, as if attacking an imaginary object. No matter how quiet it might be, however, on the least irritation, such as kicking on the slats, making any extra noise, or even when one stood in front of the cage, it would butt the slats with all the force possible. There was nothing else observable about the animal as indicating disease. There was no frothing at the mouth, and the peculiar expression so characteristic of rabies in dogs was absent. At noontime the sheep was removed from the crate and placed in a box-stall. The excitability appeared to increase, and the animal became gradually weaker. In the morning it was found lying on its side, unable to rise. There were clonic convulsions of the legs and severe opisthotonus. It needed only the touch of one's hand or of a stick on any part of the body to bring on the most aggravated convulsions. She died in one of these convulsions at 6 P.M., in the presence of Dr. Stokes and myself.

At 8 P.M. a careful autopsy was made, and cultures taken from the blood and organs. The result of the autopsy was negative. The brain was most carefully examined. Portions of the brain and medulla were reserved for rabbit inoculation. Appended will be found a description of the biological examination, made by Dr. Stokes.

In a letter of January 14th the owner says: "None have died since Friday, when four were buried. I have lost so far eighteen. The flock now looks more healthy. A gentleman who lives about four miles from here, I am informed, lost during the fall some sheep affected in the same way."

*Biological Examination.* The medulla was removed from the skull under antiseptic precautions, and a small portion of the tissue from the floor of the fourth ventricle was rubbed up with sterile water in a sterilized porcelain crucible. Two rabbits were then prepared in the following manner: The heads were shaved and a 5 per cent. solution of carbolic acid applied on cotton for about five minutes. A longitudinal incision was then made through the skin in the median line and the dura mater exposed by means of the trephine, the operation being performed under antiseptic precautions. About ten cubic centimetres of the suspension of the medulla were then injected beneath the dura of each rabbit and the wound closed.

Both animals were kept under daily observation, but nothing of interest was noted until the tenth day after the operation. Rabbit No. 1 then began to show characteristic symptoms. These symptoms began with slight incoördination of the muscles of locomotion when running. The symptoms of incoördination gradually increased, especially rapidly behind, followed by almost complete paralysis of the hind extremities, finally involving the front legs so that the animal was unable to move, in which condition he died on the eleventh day. The autopsy and the cultures of the blood and organs were negative. During the sixteenth day the second rabbit showed similar symptoms, together with clonic convulsions, and died on the seventeenth day. Autopsy and cultures were negative. (The first rabbit died during the night, so that the presence or absence of clonic convulsions was not noticed.)

The temperature of both animals during the period of incubation averaged 102°, but fell to 100° after the beginning of the attack.

*Diagnosis.* Rabies caused by transferrence of the virus of this disease from the medulla of the sheep.

*Conclusions.* The clinical symptoms of the sheep, the negative result of the autopsy, and the positive result of the inoculations point conclusively to the diagnosis of rabies. If this animal had rabies—a fact we believe beyond dispute—the other animals on the farm and on the two neighboring farms must have had the same disease.

Had inoculation experiments been made from the brain of the steers which died early in the summer the diagnosis would have been made then. This, however, would not have prevented the second outbreak of the disease, because undoubtedly the sheep died from subsequent inoculation from the bite of a rabid dog. Neither will the present knowledge of the case prevent further trouble unless something is done to check the spread of the disease in dogs. A well-bred dog is an animal most useful and companionable, but that is no reason why irresponsible people should own wild curs and allow them to run at large. A good dog-law, well enforced for a few months, would make rabies an unheardof disease. It is a disease communicable only by inoculation, and, of course, cannot arise spontaneously.

We are much indebted to Dr. N. G. Kierle, medical examiner, and to Dr. John C. Moffit, for assistance in the technique.

